# Wanting without enjoying: The social value of sharing experiences

**DOI:** 10.1371/journal.pone.0215318

**Published:** 2019-04-18

**Authors:** Eshin Jolly, Diana I. Tamir, Bethany Burum, Jason P. Mitchell

**Affiliations:** 1 Dartmouth College, Department of Psychological and Brain Sciences, Hanover, NH, United States of America; 2 Princeton University, Department of Psychology, Princeton, NJ, United States of America; 3 Harvard University, Department of Psychology, Cambridge, MA, United States of America; University of Bologna, ITALY

## Abstract

Social connection can be a rich source of happiness. Humans routinely go out of their way to seek out social connection and avoid social isolation. What are the proximal forces that motivate people to share experiences with others? Here we used a novel experience-sharing and decision-making paradigm to understand the value of shared experiences. In seven experiments across Studies 1 and 2, participants demonstrated a strong motivation to engage in shared experiences. At the same time, participants did not report a commensurate increase in hedonic value or emotional amplification, suggesting that the motivation to share experiences need not derive from their immediate hedonic value. In Study 3, participants reported their explicit beliefs about the reasons people engage in shared experiences: Participants reported being motivated by the desire to forge a social connection. Together, these findings suggest that the desire to share an experience may be distinct from the subjective experience of achieving that state. People may be so driven to connect with each other that social experiences remain valuable even in the most minimalistic contexts.

## Introduction

Why are humans such social beings? Why do we choose to vacation with friends, dine out with significant others, or watch movies with family? A rich history of psychological research suggests that people choose to be social because it satisfies an adaptive need to belong [1]. Social connection protects both physical and psychological wellbeing; in contrast, social isolation has been linked to adverse consequences such as the onset of mental health conditions including depression, anxiety, and a decline in cognitive faculties [2, 3]. In other words, establishing successful social connections provides long-term adaptive value to individuals.

However, people do not always make choices to engage in shared experiences because they are mindful of their long-term adaptive benefits. That is, rather than keeping in mind the ultimate goal of satisfying one’s need to belong [1], people often make decisions on the basis of proximate factors, like the value associated with an experience. Much in the same way that people are driven to seek food to satisfy the immediate feeling of hunger rather than a concern about long-term survival, people may choose to engage in shared experiences because of the more immediate consequences of sociality. This prompts the question: why might people immediately value shared experiences? Prior research offers several possible answers.

One possibility is that shared experiences are simply more enjoyable than solo ones. There is no dearth of aphorisms to support this idea: “Happiness quite unshared can scarcely be called happiness; it has no taste” (Charlotte Bronte); “It ain’t no fun if the homies can’t have none” (Snoop Dogg). Indeed, some studies support the intuition that shared experiences are more enjoyable. In one study for example, participants viewed a series of positive and negative images. On some trials, their friend simultaneously viewed the image; on other trials, participants alone viewed the image. Participants reported enjoying those images more when a friend simultaneously viewed the image, and show increased activation of the neural reward system including the ventral striatum and medial orbitofrontal cortex [4]. Boothby, Smith, Clark, and Bargh [5] similarly found that individuals enjoy photographs more when viewed together with a friend or familiar individual. Indeed, a recent meta-analysis of social synchrony, a particularly strong form of shared experience, showed that synchrony has a small but reliably positive impact on affect [6].

A second possibility is that the immediate value offered by sharing experiences is their ability to *amplify* emotions, making positive experiences more positive, but also negative experiences more negative. Boothby and colleagues [7, 8] for example, demonstrated that shared experiences can increase enjoyment of tasty foods and decrease enjoyment of bitter unenjoyable foods. Similarly, Shteynberg et al. [9] demonstrated that individuals co-attending to positive images find them more positive, but they also find negative images more negative, scary videos scarier, and sad videos sadder. According to this possibility, misery may love company, but sharing negative experiences may actually make them worse [10].

A third possibility is that individuals’ motivations to be social are *independent* of any consequences from sharing experiences [11]. That is, the desire for shared experiences may be robust to immediate changes in its affective qualities. If so, then people would still operate as if each opportunity to connect with another person has value, even when contexts preclude any of the typical consequences of social engagement. This possibility stems from prior work that has demonstrated two distinct components of the hedonic and motivational system: (i) wanting, or the expectancy prior to the experience that serves as a motivational force toward that experience, and (ii) liking, or the hedonic impact or affective reaction of the actual experience [12, 13, 14, 15]. Research in both humans and animals has shown that we can separately explain an organism’s *drive* or “wanting,” independent of their enjoyment, or “liking” [16]. For example, liking and wanting are supported by separate brain systems [17, 18, 19]. Activation in regions of the nucleus accumbens and striatal dopaminergic systems correspond to “wanting,” or the anticipation of a wide variety of rewards such as food or drugs [20, 21]. In contrast, medial orbitofrontal cortex and ventromedial prefrontal cortex correspond to “liking” or the subjective value associated with achieving rewarding outcomes [21]; for a review see [22].

If people *want* to share experiences, it is likely because of their robust link to social connection. However, *wanting* need not be contingent on *liking* those shared experiences. That is, the pleasure or emotional amplification derived from shared experiences may be independent of people’s proximal motivation to engage in shared experience. If sharing experiences brings long-term benefits via increasing our connection with others, our desire to share experiences should be robust to any changes in affect in the moment. A single poor dinner date should not keep us from dining with others in the future; it should also not keep us from trying to connect with others more generally. Instead, the hedonic consequences of shared experience may be dissociable from the motivational desire for shared experiences. Shared experiences can be both negative or positive *independent* of an individual’s drive to connect. This can help explain why people persist in making proximal decisions to share experiences, even if their current environment might suggest that they should not. Although individuals need not actively consider the long-term adaptive value of their social connections, the drive for social connection may be so robust, and so persistent that people will seize the opportunity to connect, even when it is not clear that those opportunities will reap positive rewards [23, 24]. Shared experiences may offer just such an opportunity. That is, people may value shared experiences even when they have little hedonic reason for doing so [11].

## Overview

This paper aimed to explore the people’s motivations for sharing experiences with others. In Study 1, we tested the hedonic impact of shared experiences. We expected that shared experiences should either increase positive affect or amplify positive and negative experiences. Based on primarily null findings from those studies, we next tested whether people’s desire to share experiences need not depend on reaping pleasure from those experiences. Finally, we tested whether people seek shared experiences, in part, because a strong motive to feel connected with others consistently buoys our desire to share experiences. If so, to the extent that people are driven to achieve social connection, and associate shared experiences with social connection, they should continue to pursue even the most minimalistic and unrewarding shared experiences. Here we tested three claims born out of this proposal.

First, Studies 1a-d tested whether shared experiences increase enjoyment or amplify emotions more generally. In these studies, we measured enjoyment during shared and solo experiences across multiple experimental contexts. We employed a variety of experimental designs, drawn from the existing literature on shared experiences, in order to identify conditions in which shared experiences do not enhance enjoyment. Participants in these studies shared both positive (1a, 1b) and negative (1c) experiences; shared experiences in the lab (1a, 1b, 1c) and online (1d); in the same room as their study partner (1b, 1c) and in different rooms from their study partner (1a); shared experiences with friends (1a, S1 Text) and with strangers who became acquainted in the laboratory (1b, 1c).

Second, Studies 2a-c tested the prediction that people will continue to choose to share experiences even in circumstances where sharing experiences does not increase enjoyment. That is, we measured if people value opportunities to share experiences even when minimalistic designs preclude shared experiences from providing any hedonic benefit. We utilized *minimalistic* shared experiences that were designed to make individuals aware of each other’s presence, while minimizing interpersonal communication, non-verbal synchrony, and spatial proximity [8]. These manipulations both limit the hedonic benefit of shared experiences and limit the possibility for engendering lasting social connection. As such, any value attributed to shared experiences in these circumstances should primarily reflect the strength of social motives. We tested if people were willing to forgo money, a stimulus with objective value, for opportunities to share experiences. The extent to which people continue to choose to share experiences, even under such circumstances, can speak to the depth of people’s drive to share experiences.

Finally, Studies 3a-b tested the prediction that this may happen because shared experiences are strongly associated with feelings of social connection. These studies explored the association between shared experiences and social connection, as a way of explaining people’s persistence in choosing to share experiences. We measured participants’ beliefs about why individuals might engage in shared experiences, what types of experiences they themselves would prefer, and what types of experiences they themselves would enjoy. These measures served to contextualize the behavior exhibited by individuals in Studies 1 and 2.

## Study 1

In Study 1 participants underwent an experience (e.g. watched a video clip) either alone or together with another individual (solo vs. shared conditions). The primary source of data collected and analyzed across these experiments was participants’ self-reported ratings of the experience or their emotional state. These design choices (i.e. self-report measures) were adapted from previous studies examining shared experiences (e.g. [7, 8, 9, 25]) and based on prior findings, we expected that participants who engage in shared experience would report higher levels of emotional intensity or increased enjoyment relative to non-shared (solo) experiences.

These studies employed a wide variety of contexts and stimuli: Study 1a employed an in-lab within-subjects design utilizing dyads of friends and enjoyable video clips; Study 1b employed an in-lab between-subjects design utilizing dyads of individuals acquainted at the lab and enjoyable video clips; Study 1c employed an in-lab between-subjects design utilizing an unfamiliar confederate acquainted in the lab, and three kinds of emotional video clips (happy, mixed emotions, and sad); Study 1d employed an online between-subjects design with enjoyable video clips. As such, these designs allow us to assess the magnitude of the effect of shared experiences on enjoyment across a wide range of design features (i.e., within subjects vs. between subjects) experimental context (i.e., in-lab vs. online), partner relationship (i.e., friends vs. strangers), or experience valence (i.e., positive vs. negative vs. mixed emotions). Details about study designs, condition specific means and standard deviations for all experiments in Study 1 are listed in Table 1. The Committee on the Use of Human Subjects at Harvard University approved this study and all following studies. All participants ages 18 or older in all studies provided informed consent. Several participants under the age of 18 (Study 1c) provided assent and consent was provided by their parental guardians.

**Table 1:** Summary overview of Study 1.

Study	Design	Content Type	Solo Rating*	Shared Rating*	Statistics [95% CI]
1a	In-lab; Within-subjects; Friends	Enjoyable Videos	*M* = 2.91 *SD* = 0.62	*M* = 2.95 *SD* = 0.60	*p* = 0.461 *d* = 0.116 [-0.20 0.45]
1b	In-lab; Between-subjects; Strangers	Enjoyable Videos	*M* = -0.01 *SD* = 0.58	*M* = -0.05 *SD* = 0.65	*p* = 0.758 *d* = 0.06 [-0.43 0.32]
1c	In-lab; Between-subjects; Confederate	Enjoyable Videos	*M* = -0.05 *SD* = 0.60	*M* = 0.06 *SD* = 0.57	*p* = 0.487 *d* = 0.12 [-0.47 0.22]
		Mixed-emotion Videos	*M* = -0.02 *SD* = 0.57	*M* = 0.05 *SD* = 0.56	*p* = 0.842 *d* = 0.04 [-0.38 0.31]
		Sad Videos	*M* = -0.01 *SD* = 0.54	*M* = 0.01 *SD* = 0.55	*p* = 0.897 *d* = 0.02 [-0.34 0.38]
1d	Online; Between-subjects; Strangers	Enjoyable Videos	*M* = 0.05 *SD* = 0.75	*M* = -0.07 *SD* = 0.95	*p* = 0.419 *d* = 0.14 [-0.47 0.19]

* Means reflect standardized composite scores for Studies 1b, 1c, and 1d

### Study 1a

#### Participants

Participants (N = 41) were native English speakers. This sample size provided at least 90% power to detect effect sizes reported by similar studies investigating this same effect [25].

#### Materials and procedure

Video clips were downloaded from YouTube (www.youtube.com) and consisted of humorous content (e.g. cute babies, children and animals, behavioral mishaps such as those featured on America’s Funniest Home Videos) and varied in length (2.14–11.91s). A separate group of 98 respondents recruited from Amazon’s Mechanical Turk service (www.mTurk.com) rated these videos on a 5-point Likert scale, ranging from “not at all” to “extremely” enjoyable. The 52 clips in this study were selected from a subset of 152 total video clips to be moderately high in enjoyment and positive affect (Mean range: 3.10–3.70).

This study utilized a within-subjects design. Pairs of friends were recruited to a lab study in which they completed a shared experience task. In this task, dyads were seated in separate rooms. Participants believed that their computers were synchronized, enabling them to watch videos concurrently. Participants then watched a series of 52 video clips across two conditions: 1) Shared: both participants watched the same video clip at the same time, and 2) Solo: each participant watched a different video clip. At the start of each trial, a cue appeared for 1000 ms to indicate in what condition the subsequent video would be played. A silhouette of one figure preceded solo trials; a silhouette of two figures preceded shared trials. Each type of silhouette was depicted in a different color (either green or purple), randomized across participants. Participants pressed the ‘1’ or ‘2’ keys during the cue to affirm they saw the cue and were aware of the condition. These cues were simply used to increase the salience of the different conditions, but responses were not analyzed. Videos for each condition were presented in randomized order and participants rated how enjoyable they found each clip using a 5-point Likert scale in a 4000 ms window after the clip finished playing (S1 Fig).

#### Results

Participants did not rate videos in the shared experience condition (*M* = 2.95, *SD* = 0.60) as more enjoyable than videos in the solo experience condition (*M* = 2.91, *SD* = 0.62), *t*(40) = 0.744, p = 0.461, *d* = 0.116, 95% CI [-0.20 0.45]. All reported confidence intervals in this and the following studies were computed on standardized effect sizes using bias-corrected and accelerated bootstrapping unless otherwise noted. Here we employed a within-subjects design, with an ecologically valid stimulus type, and participants already familiar outside of the lab, yet we found no effect of sharing minimal experiences of this type.

At least two possible design choices may explain why this study was not conducive to measuring an effect of experience sharing. First, it is possible that using a randomized within-subjects design led participants to ignore condition information as trials switched rapidly over the course of the experiment despite the use of a silhouette cue. Second, it is possible that participants did not feel as though they were sharing an experience because of the unfamiliar experimental setup (i.e., sitting in separate rooms). Study 1b was designed to replicate Study 1a, while ensuring that participants were aware of the relevant condition information.

### Study 1b

#### Participants

Participants (N = 133) were native English speakers between the ages of 18 and 25 (mean age = 22.21, *SD* = 1.16; 56% female). Participants were recruited as unfamiliar dyads who were then acquainted in the laboratory, following an approach utilized by Boothby et al. [8].

#### Materials and procedure

This study used a between-subjects design. Dyads initially entered the experiment in the same room and got to know each other by spending ten minutes asking pre-scripted questions (e.g. “What’s your favorite holiday?”) back and forth in a getting acquainted task [8]. Participants were then led into a separate lab room, where they sat side-by-side, separated by a divider that blocked their view of the other participant and his or her computer screen. Participants then watched two five-minute videos in one of two conditions explained to them by the experimenter. Participants in the shared condition watched the same videos in the same order whereas participants in the solo condition watched the videos in a different order—one watched video 1 while the other watched video 2 and vice versa. Participants in both conditions were instructed not to interact during the videos. Video 1 was an impressive live dance routine augmented by computer-programmed lighting effects. Video 2 was a touching Pixar short about the relationship between a cloud that can create life and the stork that brings that life to earth. After watching each video, participants rated how enjoyable they found the video, and answered several additional questions and measures about how they felt during the video (S1 Table).

#### Results

Although videos were not pretested for enjoyment, in response to the question “How much did you enjoy the video?” using a 7-point Likert scale (1 = “not at all”; 7 = “a great deal”), participants indicated that they found both videos quite enjoyable: video 1 (*M* = 5.96; *SD* = 1.11); video 2 (*M* = 6.07; *SD* = 1.10). To account for differences in scale ranges in the several different types of enjoyment questions participants answered (Text in S2 Table), all enjoyment ratings were standardized and combined into a composite score before being submitted to further analysis (alpha = .892). Not all participants answered each question leaving a total of 113 participants for the generation of this composite and further analysis; Studies 1b-1d used the same exclusionary criteria, namely, omitting participants who did not answer *all* questions (S2 and S3 Tables) that constitute the composite scores utilized for analysis.

Participants in the shared experience condition (*M* = -0.05, *SD* = 0.65) did not enjoy the videos more than participants in the solo experience condition (*M* = -0.01, *SD* = 0.58), *t*(109.04) = -0.308, *p* = .758, *d* = 0.06, 95% CI [-0.43 0.32]. These findings replicate the null effect from Study 1a, that sharing experiences did not affect participants’ enjoyment, suggesting that those findings are not specific to the previous experimental design, setup, and stimulus set. This study was also replicated with pairs of participants who were friends. Results remained the same (S1 Text).

Whereas Studies 1a and 1b specifically tested the effect of shared experiences on enjoyment, recent work has demonstrated that shared experiences in the form of shared attention can amplify emotional experiences more generally. Shteynberg et al., [9] found that co-attention in this way intensified feelings of sadness and happiness. Study 1c aimed to examine what effect sharing experiences has on emotional experiences by specifically including enjoyable, sad, and bittersweet video clips.

### Study 1c

#### Participants

Participants (N = 129) were native English speakers between the ages of 16 and 25 (mean age = 19.57, *SD* = 1.56; 64% female).

#### Materials and procedure

Study 1c was identical to Study 1b (between-subjects design) with two changes: 1) participants were partnered with a confederate, rather than another participant with whom they became acquainted after arriving at the lab, 2) participants viewed three new videos designed to elicit a range of emotions: happy (improvisational actors giving people high fives on an escalator), mixed emotions (an animated short depicting a kiwi bird—a variety of bird that naturally cannot fly—sacrificing its life to experience the fleeting joy of just a few moments of flight) and sadness (an elderly man recounting his experiences during the holocaust). Videos were presented in counterbalanced order. Participants answered questions about their emotional experiences consistent with each type of video (S2 Table).

#### Results

Although videos were not pretested for their emotional evocativeness, in response to the question “How did you feel while watching the video?” using a 7-point Likert scale (1 = “extremely bad”; 7 = “extremely good”), participants indicated they found each video to vary in how it made them feel: happy video (*M* = 5.63; *SD* = 0.85); mixed video (*M* = 4.33; *SD* = 1.23); sad video (*M* = 2.75; *SD* = 1.10). To account for differences in scale ranges in the several different types of questions participants answered, all ratings were standardized and combined into a composite score before being submitted to further analysis (alpha = .826; mixed-emotions video, alpha = .805; sad video, alpha = .786). Not all participants answered each question for each video leaving a subset of participants for the generation of each composite and further analysis (happy video, N = 127; mixed-emotions video, N = 126; sad video, N = 125).

Participants in the shared condition did not report feeling differently than participants in the solo condition across any of the video types (Table 1): enjoyable videos, *t*(125.07) = -0.697, *p* = .487, *d* = 0.12, 95% CI [-0.47 0.22]; mixed emotion videos *t*(124) = -0.199, *p* = .842, *d* = 0.04, 95% CI [-0.38 0.31]; sad videos, *t*(122.45) = 0.130, *p* = .897, *d* = 0.02, 95% CI [-0.34 0.38]. These results are consistent with Studies 1a and 1b. We next conducted Study 1d, to serve as a replication of Study 1b using just a single video (video 1 from Study 1b) with participants recruited from a large heterogenous online sample.

### Study 1d

#### Participants

Participants (N = 220) between the ages of 18 and 25 (mean age = 22.17, *SD* = 2.04; 44% female) were recruited from Amazon’s Mechanical Turk Labor Market [26]. This sample size provides at least 95% power to detect an effect size from Shteynberg and colleagues [9] (Study 1 GA vs alone). Due to technical errors that resulted in an exclusion of 65 participants, our effective power was reduced, but was still greater than 90%.

#### Materials and procedure

This study used a between-subjects design. Online participants were asked to choose a screen-name and avatar to represent themselves in a chat room where they would interact with another individual. This other participant was actually a set of pre-scripted computer responses. To increase credibility, there was some delay before all scripted responses appeared, with the words “response loading” displayed on the screen. Participants introduced themselves first and then saw a pre-scripted introduction from the other participant along with their purportedly chosen avatar. The pre-scripted response was designed to create common ground with most participants, and read: “hey, I'm Lauren. I'm 21 from NJ. I'm a college student. I like hanging out with friends and I'm home for the summer so just relaxing. I love music. I like taking pictures and watching movies on Netflix.” After reading introductions, participants answered three questions taken from the getting acquainted task used in Study 1b. Finally, participants were told that they were going to watch a video in a shared or solo condition as described in Study 1b; participants watched video 1 from Study 1b. During the video participants were reminded about which condition they were participating in and saw the purported other participant’s selected avatar (shared condition).

#### Results

Although the video was not pretested for enjoyment, in response to the question “How much did you enjoy the video?” using a 7-point Likert scale (1 = “not at all”; 7 = “a great deal”), participants indicated they found the video quite enjoyable: video 1 (*M* = 5.61; *SD* = 1.11). To account for differences in scale ranges in the several different types of enjoyment questions participants answered (S1 Table), all enjoyment ratings were standardized and combined into a composite score before being submitted to further analysis (alpha = .832). Unfortunately, a sizeable number of subjects indicated that the video had difficulties playing (e.g., did not load, paused in the middle). Because the shared experience condition was contingent on participants’ belief they were watching a video synchronously with another individual, playback issues would disproportionally affect this condition. For this reason, we made the decision a priori to exclude participants from *either* condition that reported technical issues. This left a total of 155 participants for the generation of this composite and further analysis.

Participants in the shared experience condition did not enjoy the video more than participants in the solo condition *t*(120.31) = -0.811, *p* = .419, *d* = 0.14, 95% CI [-0.47 0.19]. Using an online setting where participants identified themselves using aliases and avatars, sharing an experience did not change participants’ affective response to it.

### Study 1 discussion

Each of these four studies identified conditions in which participants do not find shared experience more enjoyable. These null effects were persistent to variations in experimental stimuli and design. Indeed, two additional studies were run, each using variations of these setups and stimuli (S1 and S2 Texts). These include using dyads of friends and frightening video clips. Results from these two studies fully replicate the null effects presented here. Together, these studies serve as attempts at conceptual replications of previous experiments (e.g. [7, 9]. When taken together with this earlier work, the contribution of these studies suggest that there may be boundary conditions on the extent to which people experience hedonic benefits or emotional amplification from shared experiences. Indeed, Boothby and colleagues [8] find that both spatial and social proximity moderate the effect that shared experiences have, suggesting that the current experiments may have crossed over into these boundary conditions, by providing experiences that were too minimalistic to elicit those effects. As such, we take these results to suggest that people may not *always* enjoy shared experiences more than solo ones. With that in mind, we propose that we can dissociate the hedonic benefits of shared experiences from the motivation to share experiences. By using minimalistic designs that attenuate the hedonic benefit of shared experiences (given the results from Study 1), we can test whether people will continue to pursue shared experiences. Studies 2a-c tested whether participants remain motivated to share experiences even in the context of minimalistic shared experiences with negligible hedonic benefit or emotional amplification.

## Study 2

In Study 2 we adapted the paradigm used in Study 1a to test whether participants value minimalistic shared experiences. To do so we used the paradigmatic approach of revealed preferences [27]. Participants made a series of choices that allowed us to measure how much money they were willing to forgo (if any) to share an experience with another individual. This approach has previously been used to demonstrate that macaques will forgo food in order to view high-status group-mates [28], that university students will forgo money to view attractive members of the opposite sex [29], and that people will forgo money to communicate information to others [23, 30]. Here we used this approach to test whether participants valued opportunities to share experiences with another person, more than undergoing the same experiences alone in minimalistic contexts. The experimental design allowed us to test this while continuing to minimize the influences of interpersonal communication and nonverbal synchrony.

### Study 2a

#### Participants

Participants (N = 50; 33 female) were native English speakers. This sample size provided >90% power to detect the effect size from previous research using this choice task to measure social motivations [23] and >90% power to detect the effect size of the enjoyment ratings from previous work on shared experiences [7].

#### Materials and procedure

This study was conducted in a manner similar to Study 1a, as a within-subjects design with familiar dyads. In addition to rating their enjoyment of each video, participants concurrently completed a Monetary Choice Task [30] to measure the value that individuals placed on sharing experiences.

Each member of the dyad was first separated into adjacent rooms. Participants were then told that they would be randomly assigned one of two roles, *Decider* or *Watcher*, and that their study partner would be assigned the opposite role; in actuality all subjects were always assigned the role of *Decider*. In this role, participants’ task was to make decisions to watch short video clips at the same time (Share) or at a different time (Solo) as their study partner. In the meantime, they thought their study partner would simply be watching and rating a series of videos without making any decisions.

Participants made their choices as follows: on each of 52 trials, participants were first presented with two options: *Share* or *Solo*. Each option was paired with a small monetary payoff ($0.01–0.03) for 500 ms. Payoff amounts for each choice varied randomly across trials (and were occasionally equal), as did the choice for which participants received the larger amount. This information was left on the screen for another 2000 ms during which time participants could make their decision. These choices were tallied and participants were told they would receive the money they earned throughout the study as a bonus payment upon its conclusion.

After participants made their choice, they then watched a short video clip and rated their enjoyment on a 9-point Likert scale. If participants chose the *Share* option, they were made to believe that their study partner watched the same subsequent video; if participants chose the *Solo* option, they were made to believe that their study partner watched a different video. A fixation screen ended the trial; this screen lasted for a variable duration determined by the response time of the participant, such that at least 2000 ms separated each trial from the next (S2 Fig).

Participants were always free to maximize their financial payoff by choosing whichever option paid the greater amount. However, to the extent that participants valued shared experiences, they should be willing to deviate from such economically optimal behavior and forgo money to choose the Share option. To test this hypothesis, we calculated the *point of subjective equivalence* (PSE) between the two options for each participant. This value was derived by fitting a cumulative normal distribution curve to each participant’s choices and finding the monetary value at which a participant effectively chose arbitrarily between the two trial types [23, 28]. At each value for the relative payoff difference between choosing Share and choosing Solo (-$0.02 to $0.02) we calculated the proportion of trials on which a participant chose Share. We fit a curve to these decisions using a cumulative normal distribution function defined by:

$$\frac{1}{2}\left[1+erf(\frac{x-\mu }{\sigma \sqrt{2}})\right]$$

Curve fitting was performed using constrained function minimization implemented via Nelder-Mead simplex search in Matlab (fmincon). The search was initialized at starting values of μ = 0 (constrained on the interval -2 to 2) and σ = 1 constrained on the interval (1 to 100) and ran for a maximum of 10,000 iterations. Each participant’s fitted parameters were then subjected to group level analyses. Thus, as a modeling tool, PSEs provide a quantitative metric that reflects the extent to which participants value sharing an experience relative to undergoing it alone. Specifically, it indicates the relative payoff difference at which individuals are indifferent to the two options. For example, a participant who simply wishes to maximize monetary payoff would yield a fitted PSE of 0, generating a choice curve that follows the highest value option on any given trial. In contrast, a participant who values sharing an experience with their study partner would produce a PSE significantly lower than 0, indicating a lower threshold hold for choosing to share an experience, even when it is the costly choice that means foregoing a higher payoff.

#### Results

Seven participants were excluded from all analyses because they were not native English speakers, were a research assistant on a related project, encountered technical issues when participating, or falsely represented that they knew their study partner. This left a total of 43 participants for analysis.

Results showed that participants were highly motivated to share experiences with their study partner. On average, participants chose to engage in shared experiences on 73.4% of trials. When payoff amounts were equal for both choices, participants chose to share on 86.1% of trials. Group level analysis of PSE showed that participants gave up an average of 0.996¢ (SD = 0.724¢) per trial (out of a maximum possible 2¢) to share an experience with their study partners rather than undergo an experience alone. Because PSEs were not normally distributed, a non-parametric Wilcoxon sign rank test was performed, indicating that median PSE ranks were significantly different than 0, Median = $0.724, Z = 5.676, p < .001; analyzed as a one-sample t-test yielded similar results: *t*(42) = 9.025, p < .001, *d* = 1.38, [1.12 1.64]. As a result of their decisions, participants forewent an average of 35.6% of their potential earnings in order to share an experience with their study partner.

We then tested whether participants enjoyed the shared videos more than the solo videos. To do so, we analyzed participants’ enjoyment ratings as a function of condition (Share or Solo), controlling for the money earned on a given trial, and the interaction between condition and money earned. These ratings were fit using a linear mixed model with the lme4 package [31] in R [32] specified with subject-level random intercepts and slopes for condition effects, money effects, and their interaction. P-values for linear mixed-effects models were computed using the lmerTest package [33] via Satterthwaite approximation for degrees of freedom calculations, which has been demonstrated to produce acceptable and reliable Type 1 error rates [34]. Confidence intervals were computed on model parameter estimates assuming a quadratic log-likelihood surface (Wald-method). Reported marginal means and standard deviations are centered on the mean monetary payoff level.

Despite strong preferences to share the videos, participants did not enjoy the shared videos (*M* = 5.34, *SD* = 1.17) more than the solo videos (*M* = 5.33, *SD* = 1.27), *b* = .009, *t*(115.4) = 0.07, *p* = 0.941, 95% CI of estimate [-0.24 0.25]. There was also no significant relation between participants’ enjoyment and the amount of money they earned on a particular trial *b* = -.094, *t*(463.1) = 0.66, *p* = 0.511, 95% CI of estimate [-0.37 0.19] or an interaction between money earned and condition *b* = -.208, *t*(413.4) = 1.27, *p* = 0.204, 95% CI of estimate [-0.11 0.53].

Overall results from Study 2a suggested that participants were highly motivated to engage in shared experience, as evidenced by their consistent forgoing of higher financial payoffs to do so. Participants consistently chose to share experiences even though they did not report enjoying shared experiences more than solo experience. To examine individual differences within our sample, we ran several exploratory correlation analyses to investigate the relation between participants’ sensitivity to financial payoffs and their enjoyment of their experience. These analyses revealed no significant relations (Table A in S3 Table).

### Study 2b

#### Participants

Study 2b aimed to replicate and extend the results of Study 2a with a larger sample size. Participants (N = 78; 37 female) were native English speakers. This sample size provided >90% power to detect the effect size in the choice task from Study 2a, as well as >90% power to detect the effect size of enjoyment ratings from previous work on shared experiences [7, 8].

#### Materials and procedure

Study 2b was identical to Study 2a with only two minor changes: First, participants were asked to make a single rating after all trials to indicate how much they enjoyed participating in the experiment; they made this this overall enjoyment rating using a 9-point Likert scale. This score was used to run several additional exploratory correlation analyses (Table B in S3 Table). Second, participants were provided additional instructions regarding the task. Specifically, participants were told that both individuals would ultimately watch the same set of 52 videos. The new instructions addressed one unintended interpretation of Study 2a. In Study 2a, participants may have chosen to Share a video because they wanted to watch it simultaneously with their partner, or alternatively, to ensure sure that by the end of the study, they had a large set of shared videos to discuss. As such, in Study 2b, we highlighted that their decision to Share a video would only affect whether they watched the same video at that moment; when participants chose the Solo option, participants were told that their study partner would simply watch one of the 52 videos that the participant had already seen, or had yet to see. This way if participants chose to forgo monetary rewards to share an experience, it would be unlikely that the individual had been motivated by a desire to ensure that both individuals watch the same complete set of videos.

#### Results

Nine participants were excluded due to technical malfunctions leaving a total of 69 participants for analysis. Participants were highly motivated to share experiences. On average, participants chose to engage in shared experiences on 69.6% of trials, and when payoff amounts were equal for both choices, participants chose to share 83.6% of the time. Modeling PSE showed that participants gave up an average of 0.754¢ (SD = 0.705¢) per trial to share with their study partners rather than undergo an experience alone, Median = $0.502, Wilcoxon sign-rank test, Z = 6.933, p < .001; analyzed as a one-sample t-test yielded similar results: *t*(68) = 8.89, p < .001, *d* = 1.07, [0.89 1.24]. As a result, participants forewent an average of 26.3% of their potential earnings in order to share an experience with their study partner.

Using a linear mixed effects model we again looked at the difference between participants’ enjoyment ratings between conditions, controlling for the money earned on a given trial, and the interaction between condition and money earned. Despite strong preferences to share the videos, participants did not enjoy the shared videos (*M* = 5.30, *SD* = 1.16) more than the solo videos (*M* = 5.24, *SD* = 1.15), *b* = .061, *t*(968.2) = 0.747, *p* = 0.455, 95% CI of estimate [-0.10 0.22]. There was a significant relation between participants’ enjoyment and the amount of money they earned on a particular trial *b* = .215, *t*(51.5) = 2.048, *p* = 0.046, 95% CI of estimate [0.01 0.42] but no interaction between money earned and condition *b* = -.131, *t*(98.4) = 1.13, *p* = 0.262, 95% CI of estimate [-0.36 0.10].

Using a larger sample size, Study 2b successfully replicated the results of Study 2a: participants were highly motivated to engage in shared experiences, despite not reaping any hedonic benefits from doing so.

### Study 2c

#### Participants

Study 2c aimed to replicate the findings from Studies 2a and 2b, with more sampling of participants’ choices and wider range of enjoyable videos. Participants (N = 48) were native English speakers. This sample size provided >90% power to detect the effect size in the choice task, as well as >90% power to detect the effect size of the enjoyment ratings from previous work on shared experiences [7, 8].

#### Materials and procedure

Study 2c was identical to Study 2b with two minor changes: (i) we used a new set of video clips that spanned a larger range of normed enjoyment (Mean range: 2.75–4.46), (ii) each participant completed 99 trials in order to achieve a better estimation of participant’s PSE and enjoyment ratings.

#### Results

Three participants were excluded due to technical errors leaving a total of 45 participants for data analysis. Participants were highly motivated to share experiences. On average, participants chose to engage in shared experiences on 69.5% of trials, and when payoff amounts were equal for both choices, participants chose to share 88.6% of the time. Modeling PSE showed that participants gave up an average of 0.699¢ (SD = 0.632¢) per trial to share with their study partners rather than undergo an experience alone, Median = 0.502, Wilcoxon sign-rank test, Z = 5.788, p < .001; analyzed as a one-sample t-test yielded similar results: *t*(44) = 7.412, p < .001, *d* = 1.11, [0.93 1.25]. As a result, participants forewent an average of 18.9% of their potential earnings in order to share an experience with their study partner.

Using a linear mixed effects model we again looked at the difference between participants’ enjoyment ratings between conditions, controlling for the money earned on a given trial, and the interaction between condition and money earned. Once again, participants did not enjoy the shared videos (*M* = 5.04, *SD* = 1.17) more than the solo videos (*M* = 4.90, *SD* = 1.11), *b* = .138, *t*(50.8) = 1.641, *p* = 0.107, 95% CI of estimate [-0.02 0.30]. There was no significant relation between participants’ enjoyment and the amount of money they earned on a particular trial *b* = -.034, *t*(529.1) = 0.337, *p* = 0.736, 95% CI of estimate [-0.23 0.16] and no interaction between money earned and condition *b* = .079, *t*(1084) = 0.699, *p* = 0.485, 95% CI of estimate [-0.14 0.30]. Several additional exploratory correlation analyses were also run (Table C in S3 Table).

Estimating participants’ responses with a larger number of trials yielded results consistent with Studies 2a and 2b. These findings again demonstrated that individuals were highly motivated to engage in shared experiences even in highly minimalistic scenarios, and despite affording no additional hedonic benefit.

### Study 2 discussion

Across three experiments in Study 2, individuals exhibited a strong, reliable motivation to engage in minimally shared experiences. Participants consistently chose to forgo monetary rewards to watch a video with a study partner rather than watch a video alone; on average across all experiments in Study 2, participants chose to engage in shared experiences on 69.3% of trials. At the same time, we replicated findings from Study 1 that participants did not report enjoying shared experiences more than solo experiences. These results provide evidence that individuals’ motivation to engage in shared experiences can be dissociated from the hedonic consequences of doing so. That is, individuals demonstrate strong, consistent preferences for experience sharing even if those experiences involve minimalistic contexts that don’t offer opportunities to interact or communicate and thereby limit emotional amplification or increased pleasure [8]. To try to understand participants’ social motivations, we conducted one additional set of studies that probed individuals’ beliefs about sharing experiences.

## Study 3

Studies 1 and 2 suggest that individuals do not always experience more pleasure or emotional amplification from shared experiences. Nevertheless, individuals consistently choose to engage in shared experiences–even under minimalistic conditions and even when it is costly to do so. This dissociation between wanting and liking shared experiences suggests that emotional benefits may not be the primary reason that individuals choose to engage in shared experiences. Instead we propose that the prospect of sharing experiences with others may derive from a strong affiliative motivation, one that helps foster relationships, facilitate information exchange, and improve general well-being in the long-term–all while leaving momentary affect unchanged. If so, individuals should report a strong association between sharing experiences and social connection. We test this hypothesis by probing individuals’ intuitions about the reasons people choose to share experiences, and the hedonic and social consequences of doing so.

### Study 3a

#### Participants

Participants (*N* = 404; mean age = 23.68; range: 9 years) completed the study through Amazon’s Mechanical Turk service (www.mTurk.com) and Qualtrics online survey software (www.qualtrics.com). We excluded 10 participants based on duplicate IP addresses or repeat completion of the survey. Three participants were excluded because they reported having less education than a high-school diploma. One individual was excluded because they self-reported as a non-native English speaker. This left a total of 394 participants.

#### Materials and procedure

Each participant was presented with one of three scenarios. The scenarios described experiences that mirrored Studies 2a-c. Each scenario was designed to probe three questions about why people share experiences, respectively: (1) why do people choose to share experiences? (2) why do people think *other* people choose to share experiences? and (3) how much do people think they would enjoy shared and solo experiences.

Scenario 1 asked participants (*N* = 141) to imagine that they were completing one trial of the choice task from Study 2a. They were given a choice between watching the same video clip at the same time as their friend for less money ($0.04) or watching a different video clip from their friend for more money ($0.06). Participants reported which option they would choose. Participants who chose to watch the same video clip at the same time for less money additionally reported why they made that choice.

Scenario 2 asked participants (*N* = 129) to imagine a fictitious study participant named “Amy” as she completed one trial of the choice task from Study 2a. Participants were told that Amy chose to earn less money and a watch the same video clip at the same time as her friend rather than watch a different video clip for more money. Participants then reported which of 5 options they believed best explained her choice: (1) she wanted to enjoy the video more, (2) she wanted to feel more connected to her friend while watching, (3) she wanted to have a more positive memory of the experience, (4) she wanted to invest in the friendship, or (5) she chose randomly.

Scenario 3 asked participants (*N* = 120) to imagine that they were completing one trial of the choice task from Study 2a. Participants rated which of the two choices they would enjoy more: watching the same video clip at the same time for less money or watching a different video clip for more money. Ratings were made using a 7-point Likert scale anchored from “Enjoy Share more” to “Enjoy Solo More.”

#### Results

Because Scenario 2 was presented along with possible answers on a single page, responses were filtered for submissions faster than 10 seconds, and delayed responses greater than 3 standard deviations from the mean response time. This lead to the exclusion of 6 participants in the analysis of Scenario 2.

For Scenario 1, participants believed that they would choose to watch video clips alone in order to earn more money (*N* = 123) rather than watch at the same time as a friend for less money (*N* = 18), exact binomial test against equal probabilities, *p* < .001. This result suggests that people did not expect themselves to forgo money for a shared experience, even though the participants in Study 2 overwhelmingly demonstrated this behavior. Nevertheless, we can still probe these participants’ explanations for *why* they would choose to watch a video at the same time as a friend for less money. Of the participants who did choose to share the experience, most chose to do so to feel more connected with their friend (*N* = 12), relative to the other options, Monte Carlo multinomial test against equal proportions, *p* < .001: enjoy the videos more (*N* = 2), desire a more positive memory of the experience (*N* = 1), invest in their friendship (*N* = 2), or choose randomly (*N* = 1).

For Scenario 2, participants overwhelmingly indicated that they believed Amy acted the way she did to feel more connected to her friend (*N* = 75) significantly more than any of the other options (*X*^*2*^ (4, N = 123) = 130.29, *p* < .001): enjoying the videos more (*N* = 16), desiring a more positive memory of the experience (*N* = 10), investing in her friendship (*N* = 13), or choosing randomly (*N* = 9). These results provide further evidence for the hypothesis that people are driven to share experiences by the desire to feel more connected to others

For Scenario 3, participants believed that they would experience more enjoyment from watching a video alone for more money than from watching a video with a friend for less money (*M* = 5.50, *SD* = 2.05), *t*(119) = 8.00, p < .001, *d* = 0.73 (one-sample test against mid-point of scale). In line with the findings from people’s actual experiences in Studies 1 and 2, participants overwhelmingly did *not* believe they would enjoy watching videos with a friend more than watching them alone.

Together, these findings suggest that people likely choose to share experiences for reasons other than increasing their own enjoyment. Participants did not expect that they would forgo money to share experiences. However, given the low-wage economic dynamics of the MTurk marketplace [35] it is possible these participants may over value monetary outcomes in these scenarios. We ran study 3b to address this possibility.

### Study 3b

#### Participants

Study 3b aimed to replicate the findings from Study 3a with participants who were not motivated primarily by earning money. Participants in Study 3b were college undergraduates participating for course credit. Each individual saw one of two scenarios. The scenarios were based off of Scenarios 1 and 3 from Study 3a, respectively, but edited to eliminate any mention of money. The edited scenarios were still presented as a choice between a shared and solo experience, but neither experience was associated with any monetary gain. Undergraduates psychology students (N = 262) completed this study for course credit.

#### Materials and procedure

Participants completed one of two scenarios based on those in Study 3a: (1) a non-monetary version of Scenario 1 (*N* = 136), and (2) a non-monetary version of Scenario 3 (*N* = 26). Both scenarios appeared the same as in Study 3a, but without the mention of money, thereby eliminating the influence of monetary motivations on participants’ responses.

#### Results

In Scenario 1, when money was no longer a factor, participants no longer preferred the solo option. Instead, participants predominantly believed that would choose to watch video clips with their friend (*N* = 117) rather than watch video clips alone (*N* = 19), exact binomial test against equal probabilities, *p* < .001. Of these participants, most chose to do so to feel more connected with their friend (*N* = 82), relative to the other options, Monte Carlo multinomial test against equal proportions, *p* < .001: enjoy the videos more (*N* = 14), desire a more positive memory of the experience (*N* = 6), invest in their friendship (*N* = 4), or choose randomly (*N* = 11).

In Scenario 2, even though money was no longer a factor in the decision, participants still believed that they would enjoy watching a video alone more than watching a video with a friend (*M* = 4.85, *SD* = 1.43), *t*(25) = 3.01, *p* = .006, *d* = 0.59 (one-sample test against mid-point of scale). These findings replicate those of Study 3a, and further suggest that people may not choose to share experiences solely for their own hedonic benefit.

Study 3b demonstrates that individuals’ intuitions about why people share experiences remain remarkably stable even when removing financial considerations. Specifically, participants report a strong intuition that they themselves would choose to share experiences in order to feel connected to their friends, but they do not expect any hedonic boost from doing so. If anything, participants believe they would enjoy having a solo experience more than a shared experience.

**Table 2:** Summary overview of study 3.

Study	Question	Responses	Statistics
3a	Would you prefer a shared experience for less money or a solo experience for more money?	Solo = 123 Share = 18	Exact binomial test, *p* < .001
3a	Why (if share)?	Feel more connected = 12 Enjoy more = 2 Invest in friendship = 2 More positive memory = 1 Chose randomly = 1	Montecarlo multinomial test, *p* < .001
3a	Why do you think Amy engaged in a shared experience for less money?	Feel more connected = 75 Enjoy more = 16 Invest in friendship = 13 More positive memory = 10 Chose randomly = 9	*X*^*2*^ (4, N = 123) = 130.29, *p* < .001
3a	Would you enjoy a shared experience for less money more or a solo experience for less money?	*M* = 5.50 *SD* = 2.05 (Scale: Share = 1; Solo = 7)*	*t*(119) = 8.00, *p* < .001 *d* = 0.73
3b	Would you prefer a shared experience or a solo experience?	Solo = 19 Share = 117	Exact binomial test, *p* < .001
3b	Why (if share)?	Feel more connected = 82 Enjoy more = 14 Invest in friendship = 4 More positive memory = 6 Chose randomly = 11	Montecarlo multinomial test, *p* < .001
3b	Would you enjoy a shared experience more or a solo experience more?	*M =* 4.85 *SD* = 1.43 (Scale: Share = 1; Solo = 7)*	*t*(25) = 3.01, *p* = .006 *d* = 0.59

*Values are mean ratings from 1 (share more) to 7 (solo more) for 3a Enjoyment and 3b Enjoyment. All other table values reflect participant counts.

## General Discussion

People are driven to share experiences with others. Here we investigated this drive in three lines of studies that comprised nine experiments in total. Across the seven experiments in Studies 1 and 2 and two additional variations (S1 and S2 Texts), we demonstrate circumstances in which sharing minimalistic experiences do not increase enjoyment or amplify emotions. We also found that people persist in choosing to share these minimalistic experiences, even in the absence of a hedonic boost, and at a monetary loss. In two additional studies, we found that people do not expect any hedonic benefit from sharing minimalistic experiences, and instead indicated they would choose to share experiences because of a desire for social connection.

**Fig 1:**
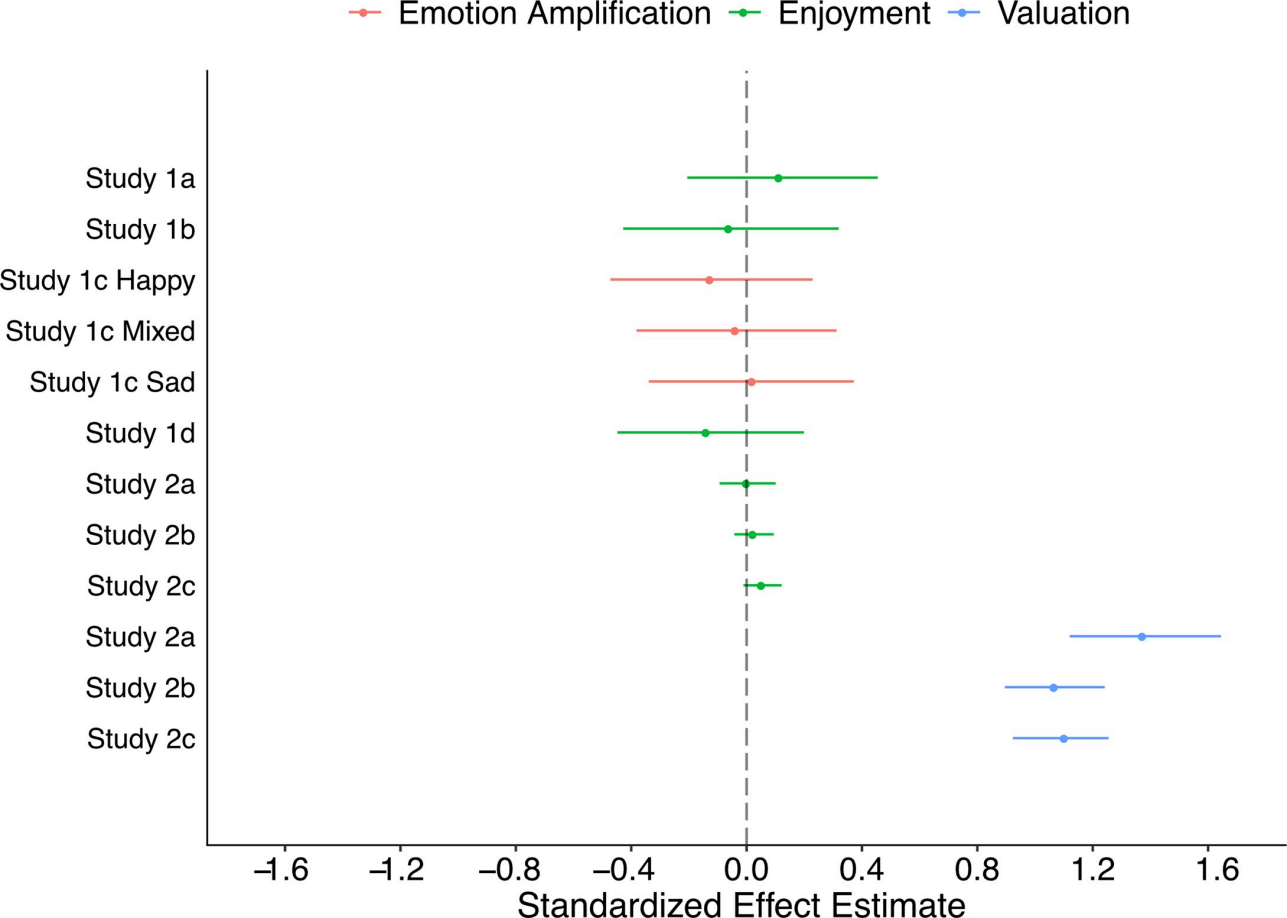
Effect sizes across Studies 1 and 2. Effect sizes for the enjoyment (green) and emotional amplification (red) of shared experiences are centered on zero, while participants effect sizes for participants’ motivation to choose shared experiences (blue) are consistently large. Standardized effect estimates: Cohen’s d (Studies 1a, 1b, 1d; Studies 2a-c, Valuation), Cohen’s d (Study 1c) and standardized regression coefficients (Studies 2a-c, Enjoyment). 95% confidence intervals were calculated via bootstrapping (Studies 1a-d, Studies 2a-c, Valuation), or by assuming a quadratic log-likelihood surface (Wald-Method; Studies 2a-c, Enjoyment).

Taken together, our results suggest that individuals are highly motivated to engage in shared experiences, even when they do not report any accompanying change in emotional experience. In Study 1, we identified conditions under which individuals did not report any increase in enjoyment, mixed emotions, or sadness when sharing an experience with another individual. This null effect persisted across a wide array of experimental variants (i.e., within and between subjects), emotional content (i.e., positive, negative, mixed, fearful, inspirational), question framings (i.e., positive and negative), social relationship types (i.e., friends and strangers acquainted in the lab) and physical proximities (i.e., same room, different room, online). Our results offer possible boundary conditions to prior work on the effects of sharing an experience on mood and emotion [7, 9, 25]. In particular, our findings comport well with findings that psychological proximity (i.e. spatial or social distance) is a strong moderator of experience sharing effects [8]: when minimally shared experiences lack opportunities for communication and interaction, the impact of shared experiences on affect may be diminished. In such minimal contexts the impact of shared experiences on enjoyment, or emotional amplification is weak at best (Fig 1). Across all studies discussed here, there is high variability in the extent to which people enjoy shared experiences, centered on an average effect size of *d* = -0.014. Although it is possible that we observed these null effects because individuals have difficulty self-reporting their internal experiences [36], prior work suggests that individuals are indeed capable of reporting positive effects of experience sharing (e.g. [7, 8, 9, 10, 25]).

Instead, our findings suggest that one can dissociate social *motivations* and emotional *experiences*. By examining individuals’ decisions in minimal contexts, we were able to ask: are individuals still motivated to engage in minimally shared experiences that do not impart a hedonic benefit? And if so why? In contrast to the highly variable, zero-centered effects observed on hedonic experience, we observed a high degree of consistency with which individuals are motivated to pursue shared experiences, centered around a large average effect size of *d* = 1.19 (Fig 1).

These results are consistent with an account of reward processing consisting of two dissociable components: “wanting” without “liking” [12, 13, 14, 17]. Whereas Studies 1 and 2 establish a null impact of minimalistic shared experiences on “liking,” Study 2 establishes that participants retain a robust “wanting” of shared experiences, even in the presence of minimal “liking.” On average in Study 2, individuals chose to share an experience approximately 71% of the time.

The value derived from shared experiences may come predominantly from social rather than hedonic motivations [11]. Study 3 suggests that individuals are motivated to share experiences for reasons other than mere enjoyment, specifically, with the goal of building social connection (Table 2). Although we don’t find differences in reported social connection in Study 1, participants in Study 3 consistently explained their own and others’ decisions to share experiences by the *desire* to feel more connected to a friend. Given humans’ strong need to belong [1], the possibility of being connected with others may be the ultimate goal that serves as the driving force of individuals’ proximate desire for shared experiences. Indeed, a desire for increased social connection has been proposed as a primary reason that individual seek to align their view of reality as closely with others as possible, which helps people both build stronger relationships and make sense of what they are experiencing [15, 37, 38]. This interpretation finds support in recent work. Boothby, Clark, and Bargh [7], find that participants sharing the experience of eating chocolate reported thinking more about the other individual during the shared experience.

This may be because participants have a strong desire to experience commonality with the inner states of other individuals. Considering how *others* feel about an experience is part of the process of establishing a “shared-reality,” which helps people to better understand the world [37, 39]. Numerous studies have noted that communicating with others’ about a topic can fundamentally alter the communicator’s memory, judgement, and impressions about that topic [40]. Similarly, observing others’ actions can influence one’s memory of their own actions [41]. For example, eating or playing sports in the presence of others can change how much individuals remember they ate or how much success they attribute to their sporting performance [42]. The idea that motivations to experience commonality with others shape thought and action is part of a broader framework known as “relevance of activated representations” (ROAR) [43]. This framework suggests that mental representations are more likely to become available and influence action based on their relevance for increasing experienced value, truth, and control [44]. In this view, experiencing commonality with others establishes a higher degree of certainty about the state of the world (increased truth), thereby becoming more available to influence thoughts, judgements, feelings, and actions.

Social connection may also explain why individuals are more expressive when sharing an experience, even when their emotional experience is unchanged. Several researchers have demonstrated that sharing an experience has *zero* effect on enjoyment, and that instead, people may simply *express* more emotion in the presence of others. For example, individuals smile more when viewing a happy video with another individual, but they do not report any concomitant increase in happiness [45] (but see [46]). That is, participants did not report enjoying the shared experience more, but they did smile more when they believed they were sharing an experience, despite being alone in a room. Similarly, people express more sadness [47] and disgust [48] in the presence of others, without experiencing parallel increases in any self-reported sadness and disgust, respectively. Given that facial displays of emotion do not always reflect an individual’s internal emotional experience [49], this work suggests that sharing experiences may influence the way in which individuals communicate with others through increased displays of emotion, while leaving subjective experience largely unaffected. Shared experiences may prompt increased social communication in order to facilitate connection, even when the opportunity to communicate isn’t currently possible.

If social connection were driven *entirely* by how good it feels to connect, any negative experiences could decrease an individual’s drive to seek future social connections. This would have dire consequence [3]. Instead, people may derive added value from social interaction, particularly for socially close individuals, in such a way that over-weights positive interactions [50]. This may serve to reinforce future collaborations and interactions with such individuals.

One explanation for why individuals chose to share experiences despite no hedonic benefit is because they relied on the general heuristic that shared experiences are more enjoyable. That is, in a wider array of uncontrolled settings outside of the laboratory, particularly where communication is present, shared experiences might indeed be more enjoyable. Using experience sampling methods Reis, O’Keefe, and Lane [51] found that individuals report experiencing higher positive affect and having more fun in social versus solitary situations. Indeed, multiple studies have shown that people enjoy experiences with other people more than experiences alone. As a result, some of these researchers have concluded that we enjoy experiences more when they are shared. But this confounds two things: (1) the enjoyment we get from the event itself (e.g., the movie we are watching) and (2) the enjoyment we get from being around other people. Real-world social situations involve communication and interaction, creating social contexts that influence emotional experiences. Individuals in the current study may fail to anticipate that sharing experiences in controlled and non-communicative experimental settings will not yield their usual hedonic benefits, simply because our participants have similarly conflated these two sources of enjoyment. Our minimalistic shared experiences—which excluded the possibility of organic interpersonal interactions and meaningful social connection—may be no match for a lifetime of shared experiences that were enjoyable, interactive, and led to social connection. For example, our participants may have underestimated the influence of communication on enjoyment, a facet deliberately eliminated in our studies. If sharing an experience is more likely to influence hedonic value or amplify emotions when people can communicate, this may help reconcile our results with some previous findings. Boothby and colleagues [8] have demonstrated that minimalistic shared experiences have moderating factors such as physical and psychological distance. Their work raises the broader possibility that there may be other such situational factors (e.g. communication) that moderate the effect that shared experiences have on individuals.

More recent theoretical developments have offered a different perspective altogether. Although individuals may have ultimate goals to establish social connections through experience sharing, they may also have ultimate goals to share information. That is, a specific component of personhood may involve the self as an agent of information [52]. In this light, the motivation to share experiences may not only be driven by a desire for social connection, but a desire to exchange experiential information with others, with the ultimate goal of facilitating decision-making and constructing a collective store of knowledge. Unfortunately, our current studies are not able to test this theory; if anything, our experiments specifically precluded information sharing between participants, diminishing the possibility that information exchange was a primary motivator of their choices. Nevertheless, the role of this ultimate motive in driving social behavior will be important to test in future experiments.

Humans are increasingly leading social lives that do not rely on in-person interactions. Online networks created virtual ecosystems in which our online identities can enact the desires of our offline identities. However, social networks such as Facebook and Twitter lack the face-to-face interactions through which we have developed our social-cognitive capacities. That is, online social media provide a novel environment for minimalistic shared experiences, possibly divorced from their typical consequences [24, 53]. In these cases, the social value of sharing experience may indeed be driven by our *desire* to connect with others and not necessarily the experiences themselves. Despite the obvious lack of in-person interaction, our social lives are increasingly influenced by others online [54]. It will be essential for future research to determine the extent to which online interactions do, or do not, forge real social connections.

Our findings help to understand the proximal drivers of human sociality. What basic ingredients of the social world do people value so deeply that they will continue to seek them out, even at cost, and with minimal positive consequences? We suggest that the hedonic effects of sharing experiences are not monolithic: the details matter, and shared experiences, even with friends, are not always more enjoyable than solo ones. This may be because we share experiences not to enhance the experiences themselves—we may be just as well off watching a movie alone as with a friend—but, ultimately, to reap the social benefits of increased connection. Shared experience may one basic social ingredient that drives our more complex social behaviors.

## Supporting information

S1 Fig Trial layout for Study 1a. Participants saw a color cue that indicated whether a video clip would be a shared experience (played together at the same time as their study partner) or a solo experience (their study partner would watch a different clip). Participants then rated their enjoyment using a 5 point Likert scale.
(TIFF)

S2 Fig Trial layout for experiments in Study 2. Participants first made a decision between watching an upcoming video clip in a share or solo experience. Each option was paired with a monetary value (0–0.03 cents). Participant’s decision determined whether the upcoming video played during a shared or solo experiences. After watching participants rated their enjoyment using a 9 point Likert scale.
(TIFF)

S1 Table Measures used for composites in Studies 1b and 1d. * This question asked participants to report how they felt while viewing the video by selecting from seven simple line drawn faces depicting gradations of emotion, ranging from a large frown (coded as the number 1) to a large smile (coded as the number 7), with a neutral face in the center (coded as the number 4).
** These questions, asked participants to circle all of the emotions that they had felt while watching the video, from a list of 20 adjectives. These emotions were coded as positive (happy, amused, delighted, intrigued, inspired, energized, entertained, in awe), negative (bored, irritated, unimpressed, and tired), or neutral (surprised, immersed, engaged, reflective, curious).
All connection questions were combined into a composite before analysis (alpha = .819). Participants did not report feeling more connected to the other participant in the shared condition (*M* = 0.07, *SD* = 0.63), than in the solo condition (*M* = -0.06, *SD* = 0.51), *t*(117) = 1.18, *p* = .242, Cohen’s *d* = 0.22.
(DOCX)

S2 Table Measures used for composites in Study 1c. * This question asked participants to report how they felt while viewing the video by selecting from seven simple line drawn faces depicting gradations of emotion, ranging from a large frown (coded as the number 1) to a large smile (coded as the number 7), with a neutral face in the center (coded as the number 4)
** These questions, asked participants to circle all of the emotions that they had felt while watching the video, from a list of 20 adjectives. Positive emotions: proud, delighted, relaxed, uplifted, impressed, hopeful, entertained, happy, warm, inspired, amused, playful, and serene; negative emotions: disturbed, somber, unsettled, sad, depressed, angry, heartbroken, and distressed; neutral emotions: alert, engaged, bored, and curious; bittersweet emotions: bittersweet, ambivalent, and conflicted. All connection questions were combined into a composite before analysis (alpha = .670). Participants did not report feeling more connected to the other participant in the shared condition (M = 0.09, SD = 0.64) than in the solo condition (M = -0.10, SD = 0.49), t(127) = -1.88, p = .063, Cohen’s *d* = 0.33.
(DOCX)

S3 Table Exploratory correlation analyses from Studies 2a-c. *Bonferroni correction for 3 non-independent comparisons.
(DOCX)

S4 Table Measures used for composite in Study S2. (DOCX)

S1 Text Sharing enjoyable experiences with friends. (DOCX)

S2 Text Sharing emotional experiences (frightening). (DOCX)

## References

[1] Baumeister R. F., & Leary M. R. (1995). The need to belong: desire for interpersonal attachments as a fundamental human motivation. *Psychological Bulletin*, 117(3), 497 7777651

[2] Heinrich L. M., & Gullone E. (2006). The clinical significance of loneliness: a literature review. *Clinical Psychology Review*, 26(6), 695–718. 10.1016/j.cpr.2006.04.002 16952717

[3] Cacioppo J. T., & Patrick W. (2009). *Loneliness*: *Human Nature and the Need for Social Connection*. W. W. Norton & Company.

[4] Wagner U., Galli L., Schott B., Wold A., Manstead A., Scherer K. R., & Walter H. (2014). Beautiful friendship: Social sharing of emotions improves subjective feelings and activates the neural reward circuitry. Social Cognitive and Affective Neuroscience, 1–29. 10.1093/scan/nsu121 PMC4448023 25298009

[5] Boothby E.J., Smith L.K., Clark M.S., & Bargh J.A. (2017). The world looks better together: How close others enhance our visual experiences. *Personal Relationships*, 24 *(*3*)*, 694–714.

[6] Mogan R., Ronald F., & Bulbulia J.A. (2017). To Be in Synchrony or Not? A Meta-Analysis of Synchrony’s Effects on Behavior, Perception, Cognition and Affect. *Journal of Experimental Social Psychology* 72, 13–20.

[7] Boothby E. J., Clark M. S., & Bargh J. A. (2014). Shared Experiences Are Amplified. Psychological Science, 25(12), 2209–2216. 10.1177/0956797614551162 25274583

[8] Boothby E. J., Smith L. K., Clark M. S., & Bargh J. A. (2016). Psychological Distance Moderates the Amplification of Shared Experience. Personality & Social Psychology *Bulletin*, 42(10), 1431–1444. 27562770 10.1177/0146167216662869

[9] Shteynberg G., Hirsh J. B., Apfelbaum E. P., Larsen J. T., Galinsky A. D., & Roese N. J. (2014). Feeling More Together: Group Attention Intensifies Emotion. Emotion, 14(6), 1102–1114. 10.1037/a0037697 25151520

[10] Shteynberg G. (2015). Shared Attention. Perspectives on Psychological Science: A *Journal of the Association for Psychological Science*, 10(5), 579–590. 10.1177/1745691615589104 26385997

[11] Tamir D.I., Hughes B.L. (2018). Social Rewards: From basic social building blocks to complex social behavior. *Perspectives on Psychological Science*, 13 *(*6*)*, 700–717. 10.1177/1745691618776263 30415630

[12] Berridge K. C., & Robinson T. E. (2003). Parsing reward. *Trends in Neurosciences*, 26(9), 507–513. 10.1016/S0166-2236(03)00233-9 12948663

[13] Berridge K. C., Robinson T. E., & Aldridge J. W. (2009). Dissecting components of reward: “liking”, “wanting”, and learning. *Current Opinion in Pharmacology*, 9(1), 65–73. 10.1016/j.coph.2008.12.014 19162544 PMC2756052

[14] Higgins E. T. (2006). Value from hedonic experience and engagement. *Psychological Review*, 113(3), 439–460. 10.1037/0033-295X.113.3.439 16802877

[15] Higgins E. T., & Pittman T. S. (2008). Motives of the Human Animal: Comprehending, Managing, and Sharing Inner States. *Annual Review of Psychology*, 59(1), 361–385. 10.1146/annurev.psych.59.103006.093726 17883333

[16] Finlayson G., King N., & Blundell J. E. (2007). Is it possible to dissociate “liking” and “wanting” for foods in humans? A novel experimental procedure. *Physiology & Behavior*, 90(1), 36–42. 17052736 10.1016/j.physbeh.2006.08.020

[17] Berridge K. C., & Kringelbach M. L. (2015). Pleasure Systems in the Brain. Neuron, 86(3), 646–664. 10.1016/j.neuron.2015.02.018 25950633 PMC4425246

[18] Miller E. M., Shankar M. U., Knutson B., & McClure S. M. (2014). Dissociating motivation from reward in human striatal activity. *Journal of Cognitive Neuroscience*, 26(5), 1075–1084. 10.1162/jocn_a_00535 24345173 PMC5808846

[19] Rademacher L., Krach S., Kohls G., Irmak A., Gründer G., & Spreckelmeyer K. N. (2010). Dissociation of neural networks for anticipation and consumption of monetary and social rewards. *NeuroImage*, 49(4), 3276–3285. 10.1016/j.neuroimage.2009.10.089 19913621

[20] Diekhof E. K., Kaps L., Falkai P., & Gruber O. (2012). The role of the human ventral striatum and the medial orbitofrontal cortex in the representation of reward magnitude—an activation likelihood estimation meta-analysis of neuroimaging studies of passive reward expectancy and outcome processing. *Neuropsychologia*, 50(7), 1252–1266. 10.1016/j.neuropsychologia.2012.02.007 22366111

[21] Knutson B., Adams C. M., Fong G. W., & Hommer D. (2001). Anticipation of increasing monetary reward selectively recruits nucleus accumbens. *The Journal of Neuroscience*: *The Official Journal of the Society for Neuroscience*, 21(16), RC159. 10.1523/JNEUROSCI.21-16-j0002.2001 PMC6763187 11459880

[22] Peters J., & Büchel C. (2010). Neural representations of subjective reward value. *Behavioural Brain Research*, 213(2), 135–141. 10.1016/j.bbr.2010.04.031 20420859

[23] Tamir D. I., Zaki J., & Mitchell J. P. (2015). Informing others is associated with behavioral and neural signatures of value. *Journal of Experimental Psychology*. *General*, 144(6), 1114–1123. 10.1037/xge0000122 26595840

[24] Tamir D.I., Ward A.F. (2015). Old Desires, New Media In Hofmann W. & Nordgren L. (Eds.), The Psychology of Desire. New York: Guilford Press.

[25] Shteynberg G., & Apfelbaum E. P. (2013). The Power of Shared Experience: Simultaneous Observation With Similar Others Facilitates Social Learning. Social Psychological and Personality Science, 4(6), 738–744.

[26] Buhrmester M., Kwang T., & Gosling S. D. (2011). Amazon’s Mechanical Turk a new source of inexpensive, yet high-quality, data? Perspectives on Psychological Science: A Journal *of the Association for Psychological Science*, 6(1), 3–5. 10.1177/1745691610393980 26162106

[27] Samuelson P. A. (1948). Foundations of Economic Analysis. Science and Society, 13(1), 93–95.

[28] Deaner R. O., Khera A. V., & Platt M. L. (2005). Monkeys pay per view: adaptive valuation of social images by rhesus macaques. *Current* Biology: CB, 15(6), 543–548. 10.1016/j.cub.2005.01.044 15797023

[29] Hayden B. Y., Parikh P. C., Deaner R. O., & Platt M. L. (2007). Economic principles motivating social attention in humans. Proceedings. Biological Sciences / The Royal *Society*, 274(1619), 1751–1756. 10.1098/rspb.2007.0368 PMC2493582 17490943

[30] Tamir D. I., & Mitchell J. P. (2012). Disclosing information about the self is intrinsically rewarding. *Proceedings of the National Academy of Sciences of the United States of America*, 109(21), 8038–8043. 10.1073/pnas.1202129109 22566617 PMC3361411

[31] Bates, D., Maechler, M., Bolker, B., & Walker, S. (2015). lme4: Linear mixed-effects models using Eigen and S4. R package version 1.1–7. 2014. Institute for Statistics and Mathematics of WU Website. http://CRAN.R-Project.Org/package=lme4. Accessed March, 18.

[32] R Core Team. R: A language and environment for statistical computing. R Foundation for Statistical Computing, Vienna, Austria (2016).

[33] Kuznetsova, A., Brockhoff, P.B. & Christensen, R.H.B lmerTest: Tests in linear mixed effects models. R package version 2.0–20. (2015).

[34] Luke S. G. (2017). Evaluating significance in linear mixed-effects models in R. *Behavior Research Methods*, 49(4), 1494–1502. 10.3758/s13428-016-0809-y 27620283

[35] Rand D. G. (2012). The promise of Mechanical Turk: how online labor markets can help theorists run behavioral experiments. *Journal of Theoretical Biology*, 299, 172–179. 10.1016/j.jtbi.2011.03.004 21402081

[36] Nisbett R.E. & Wilson T.D. (1977). Telling more than we can know: Verbal reports on mental processes. *Psychological Review*, 84(3), 231–259.

[37] Echterhoff G., Higgins E. T., & Levine J. M. (2009). Shared Reality: Experiencing commonality with others’ inner states about the world. Perspectives on Psychological Science: A Journal of the Association for *Psychological Science*, 4(5). 10.1111/j.1745-6924.2009.01161.x 26162223

[38] Festinger L. (1954). A Theory of Social Comparison Processes. Human Relations; Studies towards the Integration of the Social Sciences, 7(2), 117–140.

[39] Echterhoff G. & Higgins E.T. (2017). Creating shared reality in interpersonal intergroup communication: the role of epistemic processes and their interplay. *European Review of Social Psychology*, 28(1), 175–226.

[40] Higgins E.T. & Rholes W.S. (1978). “Saying is believing”: Effects of message modification on memory and liking for the person described. *Journal of Experimental Social Psychology*, 14, 363–378.

[41] Linder I., Echterhoff G., Davidson P.S. & Brand M. (2010). Observation inflation. Your actions become mine. *Psychological Science*, 21(9), 1291–1299. 10.1177/0956797610379860 20689054

[42] Steinmetz J., Xu, Qian, Fishbach A. & Zhang Y. (2016). Being observed magnifies action. *Journal of Personality and Social Psychology*, 111(6), 852–865. 10.1037/pspi0000065 27454927

[43] Eitam B. & Higgins E.T. (2010). Motivation in Mental Accessibility: Relevance of a Representation (ROAR) as a New Framework. *Social and Personality Psychology Compass*, 4(10), 951–967. 10.1111/j.1751-9004.2010.00309.x 21116462 PMC2992320

[44] Eitam B., Miele D.B. & Higgins E.T. (2013). Motivated Remembering: Remembering as Accessibility and Accessibility as Motivational Relevance In Carlston D.E. (Ed.), The Oxford Handbook of Social Cognition, (pp. 463–475). New York: Oxford University Press.

[45] Fridlund A. J. (1991). Sociality of solitary smiling: Potentiation by an implicit audience. *Journal of Personality and Social Psychology*, 60(2), 229–240.

[46] Hess U., Banse R., & Kappas A. (1995). The intensity of facial expression is determined by underlying affective state and social situation. *Journal of Personality and Social Psychology*, 69(2), 280.

[47] Jakobs E., Manstead A. S., & Fischer A. H. (2001). Social context effects on facial activity in a negative emotional setting. *Emotion*, 1(1), 51–69. 12894811 10.1037/1528-3542.1.1.51

[48] Williams, W.C., Nook, E., Son, J. & Zaki, J. (2014, April). Audiences enhance the expression and communication but no the experience of emotion. Poster presented at the 1st Annual Meeting of the Society for Affective Science, Washington, D.C.

[49] Ekman P. (1999). Basic emotions. *Handbook of Cognition and Emotion*, 98, 45–60.

[50] Fareri D. S., Chang L. J., & Delgado M. R. (2015). Computational substrates of social value in interpersonal collaboration. The Journal of Neuroscience: The Official Journal of the Society for Neuroscience, 35(21), 8170–8180. 26019333 10.1523/JNEUROSCI.4775-14.2015 PMC4444540

[51] Reis H.T., O’Keefe S.D. & Lane R.D. (2016). Fun is more fun when others are involved. *The Journal of Positive Psychology*, 12 *(*6*)*, 547–557. 10.1080/17439760.2016.1221123 28919919 PMC5597001

[52] Baumeister R.F., Maranges H.M. & Vohs K.D. (2017). Human self as information agent: Functioning in a social environment based on shared meanings. Review of General Psychology. 10.1037/gpr0000105

[53] Meshi D., Tamir D.I., Heekeren H.R. (2015). The emerging neuroscience of social media. *Trends in Cognitive Science*, 19 *(*12*)*, 771–782. 10.1016/j.tics.2015.09.004 26578288

[54] Kramer A. D. I., Guillory J. E., & Hancock J. T. (2014). Experimental evidence of massive-scale emotional contagion through social networks. *Proceedings of the National Academy of Sciences of the United States of America*, 111(24), 8788–8790. 10.1073/pnas.1320040111 24889601 PMC4066473

