# Supplementary figures and images for: Wanting without enjoying: The social value of sharing experiences
Source: PLoS One. 2019 Apr 18;14(4):e0215318. doi: 10.1371/journal.pone.0215318 (PMC6472755; doi:10.1371/journal.pone.0215318)

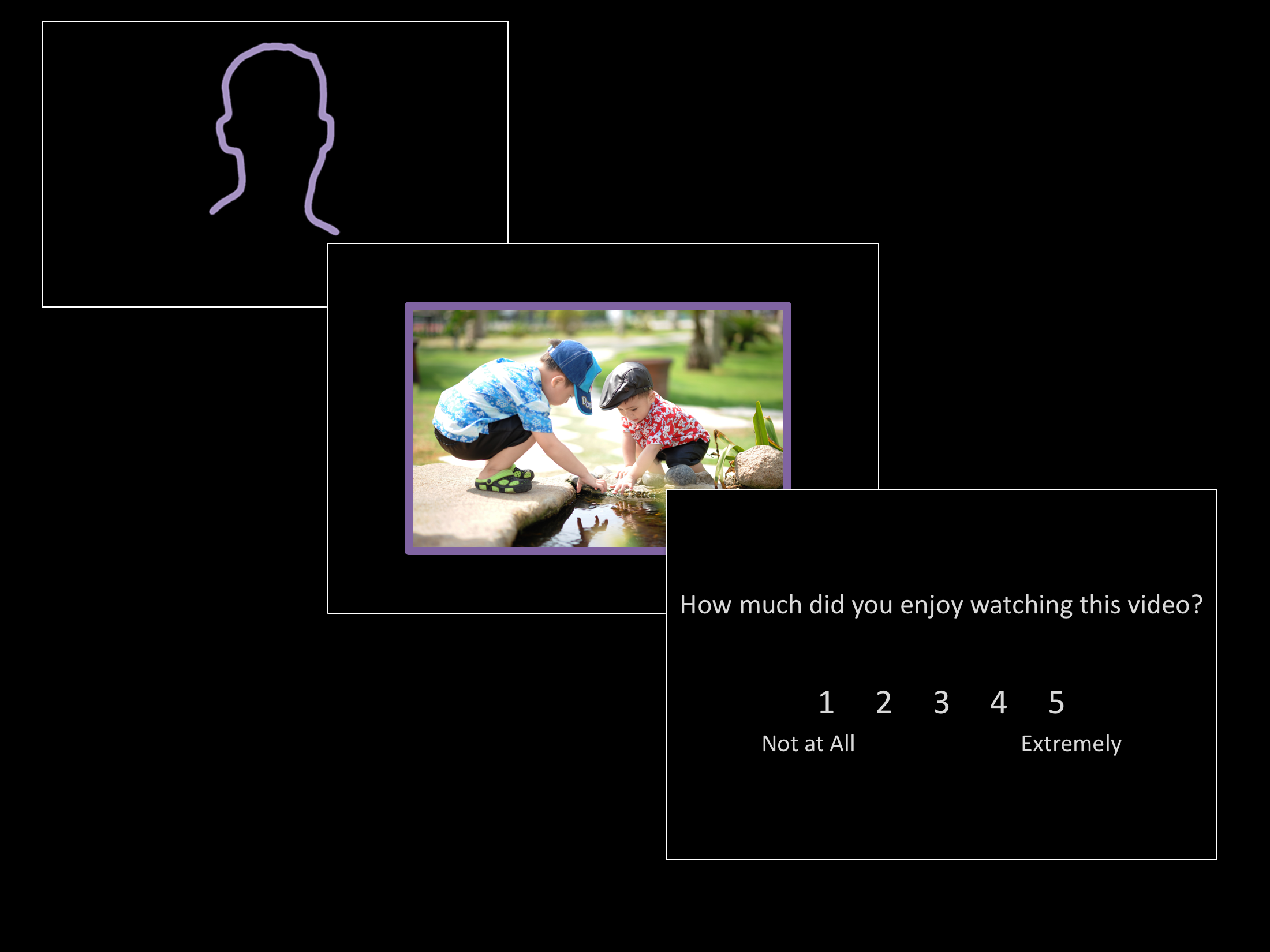

Supplement: S1 Fig — Participants saw a color cue that indicated whether a video clip would be a shared experience (played together at the same time as their study partner) or a solo experience (their study partner would watch a different clip). Participants then rated their enjoyment using a 5 point Likert scale. (TIFF) [file pone.0215318.s001.tiff]

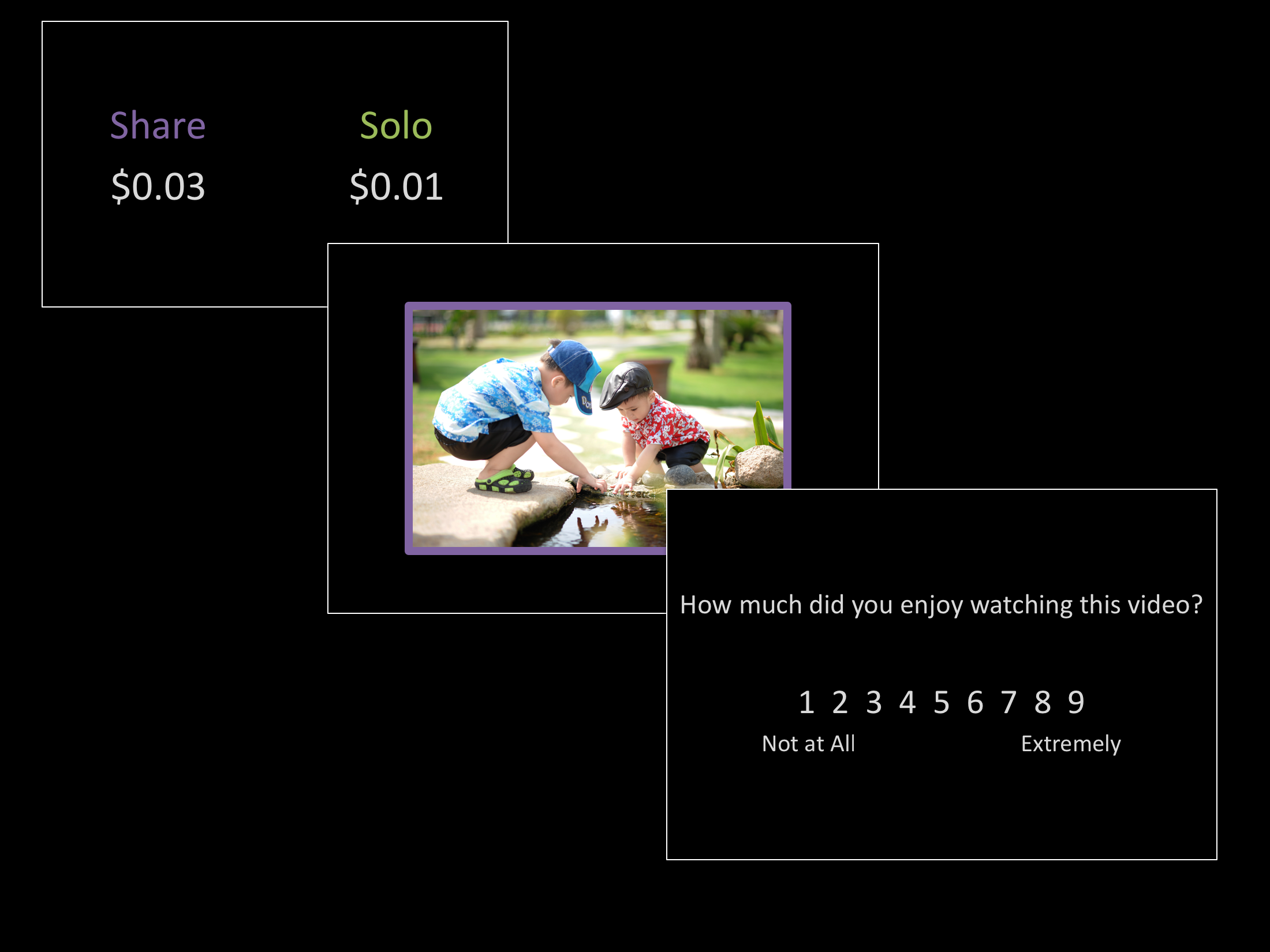

Supplement: S2 Fig — Participants first made a decision between watching an upcoming video clip in a share or solo experience. Each option was paired with a monetary value (0–0.03 cents). Participant’s decision determined whether the upcoming video played during a shared or solo experiences. After watching participants rated their enjoyment using a 9 point Likert scale. (TIFF) [file pone.0215318.s002.tiff]
