# Supplementary material for: Wanting without enjoying: The social value of sharing experiences
Source: PLoS One. 2019 Apr 18;14(4):e0215318. doi: 10.1371/journal.pone.0215318 (PMC6472755; doi:10.1371/journal.pone.0215318)
Supplement: S1 Table — * This question asked participants to report how they felt while viewing the video by selecting from seven simple line drawn faces depicting gradations of emotion, ranging from a large frown (coded as the number 1) to a large smile (coded as the number 7), with a neutral face in the center (coded as the number 4). ** These questions, asked participants to circle all of the emotions that they had felt while watching the video, from a list of 20 adjectives. These emotions were coded as positive (happy, amused, delighted, intrigued, inspired, energized, entertained, in awe), negative (bored, irritated, unimpressed, and tired), or neutral (surprised, immersed, engaged, reflective, curious). All connection questions were combined into a composite before analysis (alpha = .819). Participants did not report feeling more connected to the other participant in the shared condition (M = 0.07, SD = 0.63), than in the solo condition (M = -0.06, SD = 0.51), t(117) = 1.18, p = .242, Cohen’s d = 0.22. (DOCX) [file pone.0215318.s003.docx]

| **Question** | **Type** |
| --- | --- |
| How much did you enjoy the video? | enjoyment |
| How happy did you feel while watching the video? | enjoyment |
| How amused did you feel while watching the video? | enjoyment |
| How engaged did you feel while watching the video? | enjoyment |
| How much would you like to watch a similar video? | enjoyment |
| Which of the following faces best expresses how you felt while watching the video?* | enjoyment |
| Number of positive emotion words circles** | enjoyment |
| Number of negative emotion words circled** | enjoyment |
| Number of neutral emotion words circled** | enjoyment |
| When watching the video, how much did you think about the other participant? | connection |
| When watching the video, how connected did you feel to the other participant? | connection |
| When watching the video, how aware were you of the other participant’s presence? | connection |
| How much did the other participant affect your experience of the video? | connection |
| If you had a choice would you want to watch the same video as the other participant? | connection |
