# Supplementary material for: Wanting without enjoying: The social value of sharing experiences
Source: PLoS One. 2019 Apr 18;14(4):e0215318. doi: 10.1371/journal.pone.0215318 (PMC6472755; doi:10.1371/journal.pone.0215318)
Supplement: S2 Table — * This question asked participants to report how they felt while viewing the video by selecting from seven simple line drawn faces depicting gradations of emotion, ranging from a large frown (coded as the number 1) to a large smile (coded as the number 7), with a neutral face in the center (coded as the number 4) ** These questions, asked participants to circle all of the emotions that they had felt while watching the video, from a list of 20 adjectives. Positive emotions: proud, delighted, relaxed, uplifted, impressed, hopeful, entertained, happy, warm, inspired, amused, playful, and serene; negative emotions: disturbed, somber, unsettled, sad, depressed, angry, heartbroken, and distressed; neutral emotions: alert, engaged, bored, and curious; bittersweet emotions: bittersweet, ambivalent, and conflicted. All connection questions were combined into a composite before analysis (alpha = .670). Participants did not report feeling more connected to the other participant in the shared condition (M = 0.09, SD = 0.64) than in the solo condition (M = -0.10, SD = 0.49), t(127) = -1.88, p = .063, Cohen’s d = 0.33. (DOCX) [file pone.0215318.s004.docx]

| **Question** | **Type** |
| --- | --- |
| How sad did you feel while watching the video? | Sad |
| How sympathetic did you feel while watching the video? | Mixed |
| How depressed did you feel while watching the video? | Sad |
| How inspired did you feel while watching the video? | Mixed |
| How ambivalent did you feel while watching the video? | Mixed |
| How complex was your emotional experience while watching the video? | Mixed |
| How difficult would it be to verbalize how you felt while watching the video? | Mixed |
| How happy did you feel while watching the video? | Enjoyment |
| How amused did you feel while watching the video? | Enjoyment |
| How engaged did you feel while watching the video? | Enjoyment |
| Which of the following faces best expresses how you felt while watching the video?* | Enjoyment, Mixed, Sad |
| Number of positive emotion words circles** | Enjoyment |
| Number of negative emotion words circled** | Sad |
| Number of neutral emotion words circled** | Enjoyment, Mixed, Sad |
| Number of bittersweet emotion words circled** | Mixed |
| How connected did you feel to the other participant? | connection |
| When watching the video, how aware were you of the other participant’s presence? | connection |
