# Supplementary material for: Wanting without enjoying: The social value of sharing experiences
Source: PLoS One. 2019 Apr 18;14(4):e0215318. doi: 10.1371/journal.pone.0215318 (PMC6472755; doi:10.1371/journal.pone.0215318)
Supplement: S3 Table — *Bonferroni correction for 3 non-independent comparisons. (DOCX) [file pone.0215318.s005.docx]

**Table A.**

| **Correlation Type** | **Effect*** |
| --- | --- |
| PSE and average enjoyment | *r*(41) = 0.239, p = 0.367 |
| Percentage of trials shared and average enjoyment | *r*(41) = -0.309, p = 0.132 |
| Total earnings and average enjoyment | *r*(41) = 0.278, p = 0.217 |

**Table B.**

| **Correlation Type** | **Effect*** |
| --- | --- |
| PSE and average enjoyment | *r*(68) = 0.195, p = 0.328 |
| Percentage of trials shared and average enjoyment | *r*(68) = -0.158, p = 0.582 |
| Total earnings and average enjoyment | *r*(68) = 0.244, p = 0.131 |
| PSE and experiment enjoyment | *r*(68) = 0.144, p = 0.710 |
| Percent of trials shared and experiment enjoyment | *r*(68) = -0.036, p =.0999 |
| Total earnings and experiment enjoyment | *r*(68) = 0.205, p = .273 |

**Table C**.

| **Correlation Type** | **Effect*** |
| --- | --- |
| PSE and average enjoyment | *r*(43) = -0.096, p = 0.999 |
| Percentage of trials shared and average enjoyment | *r*(43) = 0.057, p = 0.999 |
| Total earnings and average enjoyment | *r*(43) = -0.013, p =0.999 |
| PSE and experiment enjoyment | *r*(43) = 0.212, p = 0.484 |
| Percent of trials shared and experiment enjoyment | *r*(43) = -0.227, p = 0.404 |
| Total earnings and experiment enjoyment | *r*(43) = 0.317, p = .101 |
