# Supplementary material for: Wanting without enjoying: The social value of sharing experiences
Source: PLoS One. 2019 Apr 18;14(4):e0215318. doi: 10.1371/journal.pone.0215318 (PMC6472755; doi:10.1371/journal.pone.0215318)
Supplement: S4 Table — (DOCX) [file pone.0215318.s006.docx]

| **Question** | **Type** |
| --- | --- |
| How did you feel while watching the video? | fear |
| How scared were you while watching the video? | fear |
| How unhappy were you while watching the video? | fear |
| How fearful did you feel while watching the video? | fear |
| Which of the following faces best expresses how you felt while watching the video?* | fear |
| When watching the video, how much did you think about the other participant? | connection |
| When watching the video, how connected did you feel to the other participant? | connection |
| When watching the video, how aware were you of the other participant’s presence? | connection |
| How much did the other participant affect your experience of the video? | connection |
| If you had a choice would you want to watch the same video as the other participant? | connection |
