# Supplementary material for: Wanting without enjoying: The social value of sharing experiences
Source: PLoS One. 2019 Apr 18;14(4):e0215318. doi: 10.1371/journal.pone.0215318 (PMC6472755; doi:10.1371/journal.pone.0215318)
Supplement: S1 Text — (DOCX) [file pone.0215318.s007.docx]

***Participants.*** Participants were 77 college students between the ages of 18 and 25 (mean age = 19.97, *SD* = 1.04; 49% female).

***Materials and Procedure.*** The procedure for this study was identical to the procedure of study 1b, except for several minor changes. Participants were recruited as pairs of friends and as a result did not complete the getting acquainted task. After watching both videos, participants completed the questions listed in Table S2 along with general questions about their friendship.

***Results****.* 10 participants were excluded due to technical problems, 2 who were not actually friends with each other before the study, and 4 participants who answered our understanding check question incorrectly, indicating that they were unaware of whether they were watching the videos in sync or out-of-sync with the other participant. This left 61 participants for analysis. Once again, we combined all dependent variable questions into two composites: enjoyment of the videos (alpha = .885), and perceived connection with the other participant (alpha = .786). Participants in the shared experience condition did not enjoy the videos more in the shared condition (*M* = 0.05, *SD* = 0.66) than in the unshared condition (*M* = 0.00, *SD* = 0.31), *t*(54) = -0.33, *p* = .745, Cohen’s *d* = 0.09, but felt marginally more connected to the other participant in the shared condition (*M* = 0.12, *SD* = 0.57) than in the unshared condition (*M* = -0.14, *SD* = 0.45), *t*(57) = -1.88, *p* = .066, Cohen’s *d* = 0.51, relative to participants in the unshared experience condition (analyses have different degrees of freedom because not all participants answered all questions).
