# Supplementary material for: Wanting without enjoying: The social value of sharing experiences
Source: PLoS One. 2019 Apr 18;14(4):e0215318. doi: 10.1371/journal.pone.0215318 (PMC6472755; doi:10.1371/journal.pone.0215318)
Supplement: S2 Text — (DOCX) [file pone.0215318.s008.docx]

***Participants.*** Participants were 97 unfamiliar college students between the ages of 18 and 25 (mean age = 19.10, *SD* = 2.43; 70% female).

***Materials and Procedure.*** The procedure for this study was identical to study 1b except that participants only watched one video, and it was selected to be frightening (a tense excerpt from the motion picture The Hurt Locker in which an Explosive Ordinance Disposal team attempts to diffuse a bomb strapped to an Iraqi citizen). Immediately after watching the video participants indicated how negative or positive by answering a series of questions listed in the table below.

**Results.** Nine participants were excluded because they either answered the understanding check question incorrectly, were noticeably lacking fluency in English, who suspected the confederate, or who knew the confederate. This left 88 participants for analysis. We again combined all questions into two composites: emotions during the video (alpha = .869) and perceived connection with the other participant (alpha = .722). Participants in the shared experience condition neither felt differently during the video, *t*(81) = -0.85, *p* = .401, Cohen’s *d* = 0.19, nor felt more connected to the other participant, *t*(81) = -1.83, *p* = .071, Cohen’s *d* = 0.40.
